# Serogroup Prevalence, Virulence Profile and Antibiotic Resistance of Avian Pathogenic *Escherichia coli* Isolated from Broiler Chicken

**DOI:** 10.3390/vetsci12060592

**Published:** 2025-06-16

**Authors:** Showkat A. Shah, Masood S. Mir, Shayaib A. Kamil, Majid Shafi, Mudasir A. Rather, Azmat A. Khan, Zahoor A. Wani, Sheikh Adil, Fatmah M. Alqahtani, Majid Alhomrani, Manzoor Wani

**Affiliations:** 1 Division of Veterinary Pathology, Faculty of Veterinary Sciences and Animal Husbandry, SKUAST-K, Shuhama 190006, India; vetshowkat@skuastkashmir.ac.in (S.A.S.); majidshafi321@gmail.com (M.S.); 2 Division of Veterinary Public Health, Faculty of Veterinary Sciences and Animal Husbandry, SKUAST-K, Shuhama 190006, India; 3 Division of Livestock Production and Management, Faculty of Veterinary Sciences and Animal Husbandry, SKUAST-K, Shuhama 190006, India; 4 Division of Veterinary Parasitology, Faculty of Veterinary Sciences and Animal Husbandry, SKUAST-K, Shuhama 190006, India; 5 Department of Biology, College of Science, King Khalid University, Abha 61413, Saudi Arabia; 6 Department of Clinical Laboratory Sciences, The Faculty of Applied Medical Sciences, Taif University, Taif 21944, Saudi Arabia

**Keywords:** avian pathogenic *Escherichia coli*, serogroups, virulence, genes, antibiotic resistance

## Abstract

Avian pathogenic *Escherichia coli* (APEC) causes avian colibacillosis, leading to significant economic losses and concerns for food safety in the poultry industry. This study focused on examining the virulence gene profile, antibiotic resistance prevalence, and resistance patterns of APEC isolates. A total of 250 bacterial strains were collected from birds affected by colibacillosis. Serogrouping revealed diverse serotypes, with O2 being the most common (16%), followed by O1, O8, and O76. All isolates tested positive for at minimum one virulence gene, with 7.2% carrying all five targeted genes, particularly in serogroups O1, O8, O45, and O88. The most detected gene was *iss*, present in 79.6% of isolates, followed by *tsh*, *iuc*C, *sitA*, and *pap*C. The antibiotic resistance analysis showed that all isolates exhibited multidrug resistance, although they remained susceptible to gentamicin, amikacin, ciprofloxacin, and chloramphenicol. Moreover, specific antibiotic resistance genes were known in the isolates, with *tetA* detected in 54.8%, *tetB* in 51.7%, *sul1* in 50%, and *aadA1* in 29.2%. These findings highlight the widespread antibiotic resistance in chicken carcasses, which poses a hazard to human health in terms of transfer of resistance to humans, reduced effectiveness of antibiotics and impaired ability to contain infectious diseases. Therefore, it is crucial to implement strict monitoring programs to regulate antibiotic usage in poultry production.

## Simple Summary

This study investigates avian pathogenic *Escherichia coli* (APEC), a harmful bacteria that causes colibacillosis in poultry, leading to major economic losses and potential food safety risks. We collected 250 bacterial samples from infected birds to understand how dangerous they are and how resistant they are to antibiotics. The bacteria showed a wide range of types, with serogroup O2 being the most common. All samples had at least one harmful (virulence) gene, and some had all five key genes studied. The most common gene found was *iss*, which helps the bacteria survive in the bird’s body. Antibiotic testing revealed that all APEC strains were resistant to multiple drugs, but they were still sensitive to a few, including gentamicin and ciprofloxacin. Many samples also carried genes linked to antibiotic resistance. The study highlights the need for better control of antibiotic use in poultry to protect both animal and human health.

## 1. Introduction

*Escherichia coli* is widely recognized as a normal resident of the gastrointestinal tract in both animals and humans. However, despite its routine presence, research indicates that *E. coli* can also cause a variety of illnesses in both species, affecting birds across different age groups [1]. This bacterium plays a particularly destructive role in poultry, where it is causes avian colibacillosis, a severe disease responsible for significant morbidity and mortality [2]. While chickens of all ages are susceptible to this infection, broiler chickens between the ages of 4 to 6 weeks are especially prone, often experiencing high mortality rates due to the rapid progression of the disease. Avian colibacillosis manifests as a complex syndrome, affecting various organs and tissues. Common symptoms include lesions in the air sacs (air sacculitis), inflammation of the heart lining (pericarditis), inflammation of the abdominal cavity (peritonitis), and infections in the reproductive organs, such as salpingitis [3]. These multi-organ complications underscore the severity of the disease, which can either be a primary infection or occur secondarily following other diseases, further complicating treatment efforts. The virulence of *E. coli* in poultry is primarily due to its possession of multiple virulence factors. In fact, the identification of at least five virulence-associated genes is a hallmark of avian pathogenic *E. coli* (APEC). These genes enable the bacterium to carry out critical functions like iron acquisition (*iucD and iroN*), adherence to host cells (*tsh*), toxin production (*vat*, *hlyF*), and enhancing serum resistance (*iss*, *ompT*) [4]. Together, these factors contribute to *E. coli*’s capacity to establish and sustain infections in poultry.

Interestingly, studies have shown that certain strains of avian and human extra intestinal pathogenic *E. coli* (ExPEC) share not only similar virulence genes but also a phylogenetic background [5]. This suggests a potential genetic link between strains infecting birds and humans. For example, the APEC strain O1 shares genetic similarities with human pathogens such as neonatal meningitis *E. coli* (NMEC) and uropathogenic *E. coli* (UPEC), raising concerns about the potential for horizontal gene transfer between strains [6]. Such gene transfer could lead to the spread of virulence traits between species, potentially complicating disease control efforts in both human and veterinary contexts.

In regions like Kashmir, India, the poultry industry plays a critical role in the local economy and public health. Despite the economic importance of poultry, there remains a gap in research regarding the diversity of APEC serogroups and the prevalence of virulence genes in the area [7]. While some studies have explored the presence of specific serogroups, comprehensive research on the genetic diversity of APEC strains in Kashmir is lacking. Such studies are crucial for understanding how these strains evolve and spread, particularly in the context of antibiotic resistance. In India, the misuse of antibiotics in treating bacterial infections is a pervasive issue [8]. Often, antibiotics are prescribed without first conducting an antibiogram, a laboratory test that determines the sensitivity of bacteria to different antibiotics. As a result, antibiotics may be used irrationally, exacerbating the problem of drug resistance. This is particularly problematic in the treatment of poultry diseases like colibacillosis, where the lack of data on antibiotic resistance profiles makes it difficult to implement effective treatment protocols [9]. The current study aims to address these gaps by characterizing APEC strains responsible for colibacillosis in India, with a specific focus on assessing their antibiotic resistance patterns. Understanding the prevalence of resistance genes and the specific mechanisms by which APEC strains resist antibiotics will provide valuable insights into managing and controlling the spread of colibacillosis. Additionally, this research could contribute to broader public health efforts by identifying strategies to mitigate the transmission of antibiotic-resistant bacteria from poultry to humans.

## 2. Materials and Methods

### 2.1. Location of Study and Bacterial Strains

**Figure 1:**
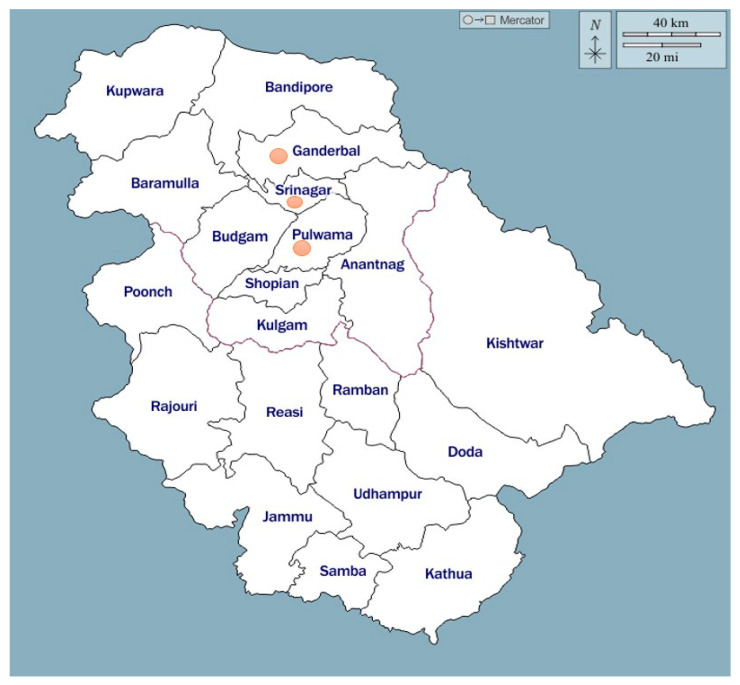
Map showing study areas from Jammu and Kashmir, India.

Samples were collected from poultry farms in the Srinagar, Pulwama, and Ganderbal districts of Jammu and Kashmir, India (Figure 1), India, involving 135 outbreaks with varying mortality rates. Suspected *Escherichia coli* outbreaks in broiler chickens were identified based on group history, clinical signs, and post-mortem lesions. Information such as flock size, mortality rate, and the total number of birds affected in each outbreak was recorded. Bacterial isolation was performed using samples from key internal organs, including the heart, intestines, lungs, liver, and spleen, at the Division of Veterinary Pathology, Faculty of Veterinary Sciences and Animal Husbandry, Sher-e-Kashmir University of Agricultural Sciences and Technology of Kashmir, Srinagar, India. A total of 250 *E. coli* isolates were obtained from broiler chickens diagnosed with colibacillosis. The samples were collected from birds that exhibited symptoms like septicemia, respiratory infections, and sudden death. Pathological findings included pneumonia, tracheitis, air sacculitis, pericarditis, peritonitis, perihepatitis, and yolk-sac infections. The identification of *E. coli* isolates was confirmed using standard morphological and biochemical tests, including Gram’s staining and Indole, Methyl Red, Voges Proskauer and Citrate (IMViC) tests, to ensure accurate diagnosis.

### 2.2. Serogrouping

The *Escherichia coli* isolates were first identified through standard morphological and biochemical tests conducted in the laboratory. These tests provided the initial characterization needed to confirm the presence of *E. coli* based on its typical physical and chemical properties. The isolates were sent to the National Salmonella and Escherichia Centre at the Central Research Institute in Kasauli, Himachal Pradesh, for further serogrouping.

### 2.3. Virulence Genotyping

All *E. coli* isolates underwent screening for specific virulence genes using polymerase chain reaction (PCR). The targeted genes included those linked to increased serum survival (*iss*), iron acquisition (*iucC* and *sitA*), temperature-sensitive haemagglutinin (*tsh*), and P fimbriae (*papC*), as detailed in Table 1. To begin the process, pure *E. coli* cultures were inoculated in nutrient broth and incubated at 37 °C. After overnight incubation, 1 mL of the culture was transferred into micro centrifuge tubes and centrifuged at 10,000× *g* for 10 min. The supernatant was discarded, and the pellet was resuspended in 100 µL of sterile PBS with gentle vortexing. The samples were then boiled for 10 min, cooled on ice for 5 min, and centrifuged again briefly. Two microliters of the resulting supernatant served as the DNA template for each PCR reaction.

The PCR reactions were prepared in sterile 0.2 mL tubes, with each reaction mixture containing 2.0 µL of the template DNA (2.5 µL for iucC), 2.5 µL of 10× buffer, 0.2 µL of 25 mM dNTP mix, 1.5 units of Taq DNA polymerase, and nuclease-free water. The concentration of MgCl_2_ was maintained at 2.5 mM. A negative control using sterilized distilled water and a positive control using an APEC isolate from serogroup O2, sourced from Sher-e-Kashmir University of Agricultural Sciences and Technology, were included in the assays.

The PCR assays were performed using a Mastercycler gradient system (Eppendorf, Hamburg, Germany). A multiplex protocol was applied for amplifying the *iss* and *iucC* genes, while a uniplex protocol was used for the remaining genes. Following amplification, the PCR products were subjected to electrophoresis on a 1% agarose gel stained with ethidium bromide. A 100 bp DNA ladder was used as a size marker. The gel was run in TAE buffer at 100 V for 1 h, and the results were visualized using the Bio Spectrum 500 Imaging System (UVP, UK).

### 2.4. Antimicrobial Susceptibility Testing

The antibiotic susceptibility of the *E. coli* isolates was evaluated in vitro using the disc diffusion method on Mueller–Hinton Agar. Each isolate was tested against a range of antibiotics, including ampicillin (10 µg), chloramphenicol (30 µg), ciprofloxacin (5 µg), doxycycline hydrochloride (30 µg), tetracycline (30 µg), amikacin (30 µg), sulfadiazine (300 µg), gentamicin (10 µg), enrofloxacin (5 µg), norfloxacin (10 µg), ofloxacin (5 µg), kanamycin (30 µg), erythromycin (15 µg), azithromycin (15 µg), and streptomycin (10 µg).

The antibiotic discs were placed on the agar plates, which were then incubated at 37 °C for 24 h. After incubation, the zones of inhibition around each disc were measured to assess the antibiotics’ effectiveness. A control plate using a standard strain of *Staphylococcus aureus* (ATCC 25923) was included to ensure the accuracy of the test. Based on the Clinical and Laboratory Standards Institute (CLSI) guidelines (CLSI M100, 31st ed., 2021) [13], the results were categorized as sensitive, intermediate, or resistant for each antibiotic tested, except for Erythromycin, where the results were interpreted as per the guidelines provided by the manufacturer (HiMedia, Mumbai, India).

### 2.5. Resistance Genotyping

*E. coli* isolates that were resistant to antibiotics such as tetracycline, sulphonamides, quinolones and streptomycin were further analyzed to detect specific antibiotic resistance genes (*tet*A, *tet*B, *aadA*1, *qnr*, and *sul1*) using PCR, as summarized in Table 2. Initially, pure *E. coli* cultures were grown in nutrient broth and incubated at 37 °C overnight. Following incubation, 1 mL of the culture was transferred into microcentrifuge tubes and centrifuged at 10,000× *g* for 10 min. The supernatant was removed, and 100 µL of sterile PBS was added to the remaining pellet. The mixture was gently vortexed, boiled for 10 min, cooled on ice for 5 min, and centrifuged again. Two microliters of the resulting supernatant were used as the DNA template for PCR reactions, which were set up in sterile tubes. Each PCR mixture contained 5.0 µL of template DNA, 2.5 µL of 10× buffer, 0.2 µL of a 25 mM dNTP mix, 1.5 units of Taq DNA polymerase, and nuclease-free water. MgCl_2_ concentration was maintained at 1.5 mM. A negative and positive control was also included in the study. APEC isolate from serogroup O2, sourced from Sher-e-Kashmir University of Agricultural Sciences and Technology, served as positive control.

The PCR assays were conducted on a Mastercycler gradient system (Eppendorf, Hamburg, Germany), with cycling conditions that included an initial denaturation at 95 °C for 3 min, followed by 35 cycles of denaturation at 94 °C for 1 min. Annealing temperatures varied according to the target gene: 57 °C for *tetA*, 56 °C for *tetB*, 58 °C for *aadA*1, 47 °C for *sul1*, and 50 °C for *qnrA*, with an extension step at 72 °C for 1 min. The PCR concluded with a final extension at 72 °C for 10 min. To analyze the amplified DNA, the products were run on a 1% agarose gel stained with ethidium bromide, along with a 100 bp DNA ladder for size comparison. The gel was subjected to electrophoresis in TAE buffer for 1 h at 100 V, and the results were visualized using the BioSpectrum 500 Imaging System (UVP, California, UK).

### 2.6. Statistical Analysis

The data analysis was performed using the chi-square test with the MedCalc software (19.2.6 version). Fisher’s exact test and the chi-square test with Yates’ correction were applied to assess the significance of correlations between two genes, following the methodology described [17].

## 3. Results

### 3.1. Samples

A total of 135 colibacillosis outbreaks in broiler chickens across various age groups were reported from three districts: Srinagar (n = 38), Ganderbal (n = 60), and Pulwama (n = 37). The overall mortality rate observed was 3.1%, with the highest mortality of 4.6% occurring in the 8–14-day age group, while the lowest rate of 1.1% was noted in chickens older than 29 days. Among 4255 necropsied carcasses, 1088 were diagnosed with colibacillosis, resulting in a case prevalence of 25.6%. The proportionate mortality attributed to colibacillosis was highest in the 15–21-day age group, accounting for 36.1% (397 out of 1088 cases), whereas the lowest mortality rate of 4.3% (47 out of 1088 cases) was recorded in chickens older than 29 days. These findings highlight the significant impact of colibacillosis on broiler chickens, particularly in younger age groups.

### 3.2. Serogrouping

Among the 250 avian pathogenic *E. coli* isolates, a total of 14 different serogroups were identified (Table 3). Notably, the O antigen for 23.2% of the isolates could not be determined, classifying them as untypeable (UT). Of the isolates that were successfully typed, the most prevalent serogroups included O2 (16%), O1 (12.4%), O8 (11.6%), and O76 (9.2%). Furthermore, seven isolates (2.8%) were categorized as rough.

### 3.3. Virulence Genes

**Figure 2:**
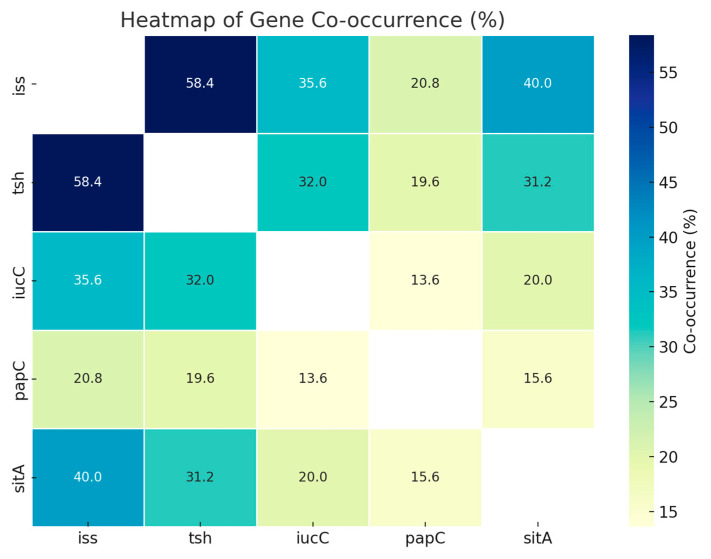
Heatmap showing the pairwise co-occurrence percentages of five virulence-associated genes (*iss, tsh, iucC, papC,* and *sitA*) among the bacterial isolates. The color intensity represents the percentage of isolates positive for both genes in each pair. Higher co-occurrence is indicated by darker shades of blue. Notably, the iss and tsh gene pair showed the highest co-occurrence (58.4%), followed by *iss* and *sitA* (40.0%). Blank diagonal cells represent self-comparisons and are not applicable.

Identifying virulence factors is crucial for understanding the pathogenic mechanisms of disease. Table 4 shows the prevalence percentages of isolates (n = 250) that tested positive for each virulence gene. For example, 79.6% of the isolates tested positive for iss, indicating that 199 of the 250 isolates included this gene. The relationships among these genes were further analyzed using the Chi-square test and Fisher’s exact test with Yates’ correction, as shown in Table 5 and Figure 2. A total of 146 strains (58.4%) tested positive for both *iss* and *tsh*, while 77 strains (30.8%) had either *iss* or *tsh*. Notably, 23 strains (9.2%) did not carry either gene.

### 3.4. Antibiotic Resistance

**Figure 3:**
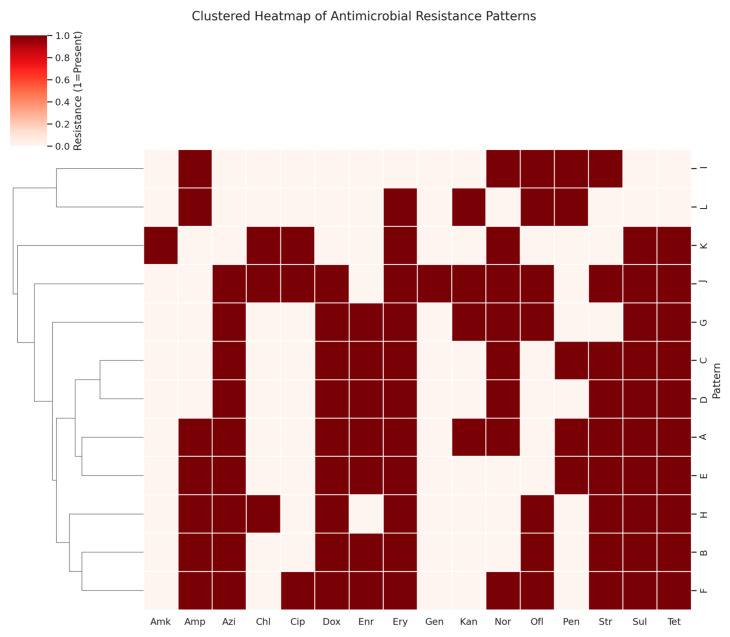
Clustered heatmap of antimicrobial resistance profiles among 250 avian pathogenic *E. coli* isolates. Each row represents a distinct resistance pattern (A–L), and columns represent individual antibiotics. Red cells indicate resistance (value = 1). Hierarchical clustering was applied to group patterns based on similarity, allowing identification of severity clusters. Patterns J, F, and H exhibited the broadest resistance spectrum, while patterns I and L showed relatively narrow resistance profiles.

A total of sixteen antibiotics were employed to determine the antibiogram for 250 *E. coli* isolates, with the resistance profiles summarized in Table 6. A significant level of antibiotic resistance was observed, as all isolates displayed resistance to multiple antibiotics. The majority of the isolates showed sensitivity to gentamicin, amikacin, ciprofloxacin, and chloramphenicol. Notably, each isolate was resistant to at least five different antibiotics, resulting in twelve distinct resistance patterns (Table 7, Figure 3). The most prevalent resistance pattern (Pattern A) was found in 43 isolates, which showed resistance to a range of antibiotics including tetracycline, ampicillin, streptomycin, kanamycin, doxycycline, erythromycin, azithromycin, norfloxacin, enrofloxacin, and sulfadiazine. Conversely, the least common resistance pattern (Pattern L) was observed in only eight isolates. The highest resistance rates were against erythromycin (94.8%), tetracycline (92%), sulfadiazine (92%), doxycycline (88%), azithromycin (88%), streptomycin (86.4%), enrofloxacin (77.6%), and norfloxacin (67.6%). In contrast, low resistance levels were noted for amikacin (4%), gentamicin (4%), chloramphenicol (14.4%), and ciprofloxacin (15.6%). Overall, 88% of isolates were resistant to eight or more antimicrobials, with no isolates being sensitive to all sixteen antibiotics tested or resistant to all of them. Antimicrobial therapy remains the primary method for controlling APEC-induced colibacillosis. However, a troubling trend of antibiotic resistance was observed in this study, with *E. coli* isolates showing high levels of resistance to common antibiotics such as erythromycin (94.8%), tetracycline (92%), sulfadiazine (92%), doxycycline (88%), and azithromycin (88%). Resistance to streptomycin (86.4%), enrofloxacin (77.6%), and enrofloxacin (67.6%) was also common, with many strains displaying multi-drug resistance patterns.

Isolates that exhibited phenotypic resistance to tetracycline, sulfadiazine, streptomycin, and ciprofloxacin were further screened for specific antibiotic resistance genes, including *tetA* and *tetB*, *sul1*, *aadA1*, and *qnrA*, respectively. Among the 230 tetracycline-resistant isolates, *tetA* was detected in 126 (54.8%) and *tetB* in 119 (51.7%). All tetracycline-resistant isolates also displayed resistance to sulfadiazine, with *sul1* identified in 115 (50%) of these isolates. For the 216 streptomycin-resistant isolates, the *aadA1* gene was found in 63 (29.2%) of them. However, the *qnrA* gene was not detected in any of the ciprofloxacin-resistant isolates, as indicated in Table 8.

## 4. Discussion

In this study, colibacillosis was responsible for 25.6% of the total mortality in broiler chickens, making it a significant factor in the health challenges facing the poultry industry [18]. Our analysis revealed substantial serological diversity among *Escherichia coli* isolates, with serogroup O2 being the most frequent at 16%, followed by O1 (12.4%), O8 (11.6%), and O76 (9.2%). This range of serogroups aligns with previous studies, which also reported O2 as a common serogroup among poultry isolates [19]. However, some research points to O78 as the most prevalent serogroup, highlighting geographical variations [20]. The finding that over half of the isolates did not belong to major serogroups underscores the wide diversity of *E. coli* strains linked to colibacillosis, suggesting that serotyping alone may not be sufficient for identifying APEC [21].

Research on APEC has increasingly focused on pathogenesis and molecular epidemiology, demonstrating that these strains carry a variety of virulence genes that are critical for causing disease [22]. These virulence factors serve as molecular markers that help in diagnosing and managing APEC infections, ultimately reducing poultry production losses [23]. In this study, five key virulence genes were examined in 250 *E. coli* isolates. The *iss* gene, known to enhance bacterial survival in the host’s bloodstream, was the most common, found in 79.6% of isolates [24]. These findings corroborate with the results of other researchers who reported the iss gene to be significantly more prevalent in APEC in chickens from Brazil [25] and Ireland [26], respectively. In the present study, the most frequent genes encountered in APEC isolates were genes that help the bacteria sustain in circulation, organs that include genes for serum survival (*iss*), which corroborated the findings of other studies [27]. Additionally, *tsh*, which contributes to tissue colonization, was present in 71.2% of isolates. Around 60% of the *iss*-positive isolates also carried *tsh*, indicating that these strains could be primary pathogens.

The *papC* gene, which facilitates adhesion to internal organs and offers protection against immune responses, was detected in 29.2% of isolates. This is partially consistent with earlier studies, where the prevalence of *papC* was reported at 40.4% [27]. The aerobactin gene *iucC*, part of the iron acquisition system, was found in 29.2% of isolates. In contrast, other studies [28] reported a much higher prevalence of *iucC* (77.5%) in APEC strains. Aerobactin plays a critical role in allowing *E. coli* to thrive in iron-limited environments, such as those found within a host, and is vital for APEC’s pathogenicity. Another iron transport-related gene, *sitA*, was identified in 46% of isolates. The sitABCDE system aids in the transport of iron and manganese, supporting bacterial survival under oxidative stress [29].

In poultry production, antimicrobial drugs are widely employed and typically added to the feed or drinking water. Antimicrobials are used to treat illnesses and promote growth, prevention and management of illnesses, such as colibacillosis in the poultry sector. However, for treatment to be effective and to prevent drug resistance in clinical bacterial isolates, antimicrobials must be used sparingly. This can be accomplished by first determining the clinical bacterial isolates’ in vitro antibiotic sensitivity test results [21]. In our study, the lowest resistance rates were observed for amikacin (4%), gentamicin (4%), chloramphenicol (14.4%), and ciprofloxacin (15.6%), which is consistent with findings from Zimbabwe [30]. This highlights the complex interplay between phenotypic resistance and the presence of specific resistance genes among the *E. coli* isolates studied.

To better understand the mechanisms behind this antibiotic resistance, PCR was conducted on phenotypically resistant isolates. The tetracycline resistance genes *tetA* and *tetB* were found in 54.8% and 51.7% of isolates, respectively, indicating a strong correlation between phenotypic resistance and the presence of resistance genes. For sulfadiazine resistance, the *sul1* gene was detected in 50% of resistant isolates. This finding matches earlier studies that highlighted geographical differences in the prevalence of resistance genes, often influenced by local antibiotic use practices [30]. The *aadA1* gene, which confers resistance to streptomycin, was present in 29.2% of isolates, a figure comparable to the 68.4% prevalence reported by other researchers [31]. Interestingly, the *qnr* gene, which is linked to resistance to fluoroquinolones such as ciprofloxacin, was not detected in any of the ciprofloxacin-resistant isolates. When the *qnr* gene is absent, bacteria must rely on other strategies to resist ciprofloxacin-like mutations in quinolone resistance-defining regions (QRDRs) which alter the structure of DNA gyrase and topoisomerase IV, making them less susceptible to ciprofloxacin’s binding and efflux pump overexpression that actively pump antibiotics out of the cell, reducing the intracellular concentration of the drug [32]. Previous studies have reported a *qnr* prevalence of 36.8% [33], indicating that resistance mechanisms can vary widely across different regions and bacterial populations. In the present study, though the presence of resistance genes such as tetA, tetB, and sul1 was associated with reported phenotypic resistance, it is crucial to highlight that our molecular screening only included a subset of known resistance genes. For example, sulfonamide resistance could include sul2 or sul3, which were not investigated. Similarly, resistance to tetracyclines and aminoglycosides may be mediated by other gene families, and fluoroquinolone resistance in E. coli is frequently associated with point mutations in the QRDR of gyrA and parC, which were not tested in this work. As a result, our molecular data provide only a partial picture of the resistance mechanisms implicated.

The World Health Organization (WHO) and the World Organization for Animal Health (WOAH) have expressed global concerns about the rising rate of antimicrobial resistance (AMR), which is consistent with the findings of this study. Due in large part to the overuse of antibiotics in both the human and animal sectors, both organizations rank AMR as one of the major dangers to global health. WHO’s Global Action Plan on AMR [34] and WOAH’s Strategy on Antimicrobial Resistance [35] highlight the necessity of more stringent regulatory monitoring and antibiotic stewardship initiatives, especially in the poultry industry. This study’s identification of multidrug-resistant *E. coli* bacteria confirms these worldwide concerns and highlights the pressing need for alternate strategies, including strategies such as immunization, biosecurity, and enhanced diagnostics, to combat AMR in the poultry industry.

## 5. Conclusions

In conclusion, our study identified a diverse range of virulence genotypes among avian pathogenic *Escherichia coli* (APEC) strains, contributing to the understanding of the distribution of these virulence-associated genes. This knowledge is crucial for advancing epidemiological research and understanding the mechanisms underlying colibacillosis in poultry. Additionally, the high prevalence of multiple antibiotic resistance observed in *E. coli* isolates from poultry in Kashmir emphasizes the growing concern of antimicrobial resistance in veterinary medicine. The findings from this study underscore the importance of in vitro antimicrobial susceptibility testing for avian *E. coli* isolates, which could provide essential guidance to veterinarians in selecting appropriate and effective antimicrobial treatments. Establishing routine antimicrobial surveillance programs is critical, not only to manage resistance in animal populations but also to monitor the potential transmission of resistance genes, particularly through plasmids, from veterinary sources to human pathogens. This cross-sectoral approach is key to mitigating the spread of antibiotic resistance and preserving the efficacy of antimicrobial agents in both animal and human health contexts. The reported genetic resistance patterns in this study reflect just a fraction of the genes tested and may not represent the entire range of resistance-imparting mechanisms. Further research employing whole-genome sequencing or larger PCR panels is warranted.

## Figures and Tables

**Table 1:** Details of primers used in virulence gene study along with amplicon size.

S.No.	Target Gene	Sequence 5′–3′	Primer Conc. (µM)	Product Size	Reference
1	*iss*	(F)GTGGCGAAAACT AGTAAAACAGC	1.0	762	[10]
		(R)CGCCTCGGGGTGGATAA			
2	*iucC*	(F) CGCCGTGGCTGGGGTAAG	1.0	541	[11]
		(R) CAGCCGGTTCAC CAAGTATCACTG			
3	*sitA*	(F)AGGGGGCACAACTGATTCTCG	0.5	608	[12]
		(R)TACCGGGCCGTTTTCTGTGC			
4	*tsh*	(F) GGTGGTGCACTGGAGTGG	1.0	642	[10]
		(R) AGTCCAGCGTGATAGTGG			
5	*papC*	(F) GTGGCAGTATG AGTAATGACCGTTA	0.5	205	[10]
		(R) ATATCCTTTCTGC AGGGATGCAATA			

**Table 2:** Details of primers used in antibiotic resistance gene study along with amplicon size.

Antimicrobial Agent	Resistance Gene	Sequence	Product Size	Primer Conc.	Reference
Quinolones	*qnrA*	(F) GGT ATG GAT ATT ATT GAT AAA G (R) CTA ATC CGG CAG CAC TAT TTA	670	0.2	[14]
Sulfonamide	*Sul1*	(F) TTC GGC ATT CTG AAT CTC AC (R) ATG ATC TAA CCC TCG GTC TC	822	0.2	[15]
Streptomycin	*aadA1*	(F) TAT CCA GCT AAG CGC GAA CT (R) ATT TGC CGA CTA CCT TGG TC	447	0.2	[15]
Tetracycline	*tet(A)*	(F) GGT TCA CTC GAA CGA CGT CA (R) CTG TCC GAC AAG TTG CAT GA	577	0.2	[16]
	*tet(B)*	(F) CCT CAG CTT CTC AAC GCG TG (R) GCA CCT TGC TGA TGA CTC TT	634	0.2	[16]

**Table 3:** Serogrouping diversity in *E. coli* isolated from colibacillosis-affected broiler chicken.

S. No.	Serogroup	Number of Isolates	Percentage ^A^
1.	O2	40	16.0 ^g^
2.	O1	31	12.4 ^fg^
3.	O8	29	11.6 ^efg^
4.	O76	23	9.2 ^ef^
5.	O114	11	6.66 ^de^
6.	O45	10	4.0 ^cd^
7.	O26	09	3.6 ^bcd^
8.	O20	08	3.2 ^abcd^
9.	O89	08	3.2 ^abcd^
10.	R	07	2.8 ^abc^
11.	O59	04	1.6 ^abc^
12.	O88	04	1.6 ^abc^
13.	O101	03	1.2 ^ab^
14.	O11	03	1.2 ^ab^
15.	O126	02	0.8 ^a^
16.	UT	58	23.2 ^h^
Total		250	

^A^ Values followed by different letters are significantly different (*p* < 0.05).

**Table 4:** Prevalence of virulence genes in avian pathogenic *E. coli* isolates across serogroups.

Serogroup	Virulence-Associated Gene					No. of Isolates
	*iss*	*tsh*	*iucC*	*papC*	*sitA*	
O2	+	+	+	-	-	16
	+	+	-	-	+	12
	+	-	+	-	+	6
	-	-	+	+	+	2
	+	-	-	-	-	2
	-	+	-	-	+	2
O1	+	+	+	+	+	4
	+	+	-	-	+	5
	+	+	-	+	+	8
	-	+	+	+	-	3
	-	-	-	+	+	2
	+	-	+	-	-	9
O8	+	+	+	+	+	5
	+	+	+	-	-	6
	+	-	-	-	+	3
	-	-	-	+	-	4
	-	+	+	+	-	3
	+	+	-	-	-	8
O76	+	+	-	-	-	6
	+	+	+	-	+	4
	+	-	+	+	-	3
	-	+	-	+	-	2
	-	-	+	-	+	3
	+	+	-	-	+	5
O114	+	+	+	-	-	2
	+	+	-	-	-	2
	+	-	-	-	+	2
	+	+	-	+	-	3
	+	+	+	+	-	2
O45	+	+	+	+	+	2
	+	-	-	+	+	2
	+	-	+	+	-	2
	-	+	+	-	-	2
	+	+	-	-	+	1
	+	-	-	-	-	1
O26	+	+	+	-	-	2
	+	+	-	-	-	1
	+	+	-	-	+	2
	-	-	+	+	+	1
	-	+	+	-	-	1
	+	-	-	+	+	2
O20	+	+	-	-	+	2
	+	+	+	-	-	1
	+	-	-	-	+	1
	-	-	+	-	+	1
	+	+	-	-	-	2
	+	-	+	-	-	1
O89	+	+	+	-	-	1
	+	+	-	-	-	1
	+	+	-	-	+	2
	-	+	-	-	-	2
	+	-	-	+	+	2
O59	+	+	+	-	+	1
	+	+	-	-	-	1
	+	+	-	-	+	1
	+	+	+	-	-	1
O88	+	+	+	+	+	1
	+	+	-	-	-	1
	+	+	-	+	-	1
	+	-	-	-	-	1
O101	+	+	+	-	-	1
	+	+	+	-	+	1
	+	+	-	-	+	1
O11	+	+	-	-	+	1
	+	-	+	-	+	1
	-	+	+	-	+	1
O126	+	+	+	-	+	1
	+	+	+	-	-	1
Rough	+	+	+	-	-	2
	+	+	-	-	-	1
	+	-	-	+	+	1
	+	+	-	-	+	1
	-	-	+	-	+	1
	+	+	+	-	+	1
UT	+	+	+	+	+	6
	+	+	+	-	+	4
	+	+	-	-	-	8
	+	-	-	+	-	3
	-	-	-	-	-	3
	-	+	-	-	-	3
	+	-	+	-	+	4
	+	+	-	-	+	4
	-	+	+	-	-	5
	-	-	-	-	+	3
	-	-	+	-	-	3
	+	-	-	-	-	3
	-	+	-	+	-	4
	+	+	-	+	-	5
TOTAL NO.	199	178	117	73	115	250
TOTAL %	79.6	71.2	46.8	29.2	46.0	

**Table 5:** Percentage of strains with the given pair of virulence-associated genes among 250 avian pathogenic *E. coli* strains.

	*iss*	*tsh*	*iucC*	*papC*	*sitA*
*iss*	**-**				
*tsh*	58.4 *	-			
*iucC*	35.6	32.0 *	-		
*papC*	20.8	19.6	13.6	-	
*sitA*	40.0 *	31.2	20.0	15.6	**-**

The percentage of strains harboring both genes is indicated. * p ≤ 0.05 (strong association between genes).

**Table 6:** Antibiotic susceptibility profiles of 250 *E. coli* isolates from chicken with colibacillosis.

Antibiotic	Disc Content (µg)	Susceptible No. (%)	Intermediate No. (%)	Resistant No. (%)
Penicillins				
Ampicillin (Amp)	10	75 (30)	59 (23.6)	156 (62.4)
Aminoglycosides				
Amikacin (Amk)	30	217 (86.8)	23 (9.2)	10 (4)
Gentamicin (Gen)	10	225 (90)	15 (6)	10 (4)
Kanamycin (Kan)		145 (58)	28 (11.2)	77 (30.8)
Streptomycin (Str)	10	34 (13.6)	0	216 (86.4)
Tetracyclines				
Tetracycline (Tet)	30	5 (2)	15 (6)	230 (92)
Doxycycline (Dox)	30	20 (8)	10 (4)	220 (88)
Macrolides				
Erythromycin (Ery)	15	13 (5.2)	0	237 (94.8)
Azithromycin (Azi)	15	20 (8)	10 (4)	220 (88)
Fluoroquinolones				
Ciprofloxacin (Cip)	5	168 (67.2)	43 (17.2)	39 (15.6)
Norfloxacin (Nor)	10	60 (24)	21 (8.4)	169 (67.6)
Ofloxacin (Ofl)	5	75 (30)	58 (23.2)	117 (46.8)
Enrofloxacin (Enr)	5	30 (12)	26 (10.4)	194 (77.6)
Chloramphenicol (chl)	30	139 (55.6)	75 (30)	36 (14.4)
Sulfonamides				
Sulfadiazine (Sul)	300	10 (4)	10 (4)	230 (92)

**Table 7:** Antimicrobial resistance patterns of 250 avian pathogenic *E. coli* isolates.

Pattern	No. of Isolates	Resistance Pattern
A	43	Tet, Amp, Str, Kan, Dox, Ery, Azi, Nor, Enr, Sul
B	35	Tet, Amp, Str, Dox, Ery, Azi, Ofl, Enr, Sul,
C	30	Tet, Str, Dox, Ery, Azi, Nor, Enr, Sul
D	27	Tet, Str, Dox, Ery, Azi, Nor, Enr, Sul
E	23	Tet, Amp, Str, Dox, Ery, Azi, Enr, Sul
F	19	Tet, Amp, Str, Dox, Ery, Azi, Nor, Ofl, Enr, Sul, Cip
G	17	Tet, Kan, Dox, Ery, Azi, Nor, Enr, Sul, Ofl
H	16	Tet, Amp, Str, Dox, Ery, Azi, Chl, Sul, Ofl
I	13	Amp, Str, Nor, Ofl
J	10	Tet, Str, Kan, Dox, Ery, Azi, Nor, Ofl, Chl, Sul, Cip, Gen
K	10	Tet, Ery, Nor, Chl, Sul, Cip, Amk
L	7	Amp, Kan, Ery, Ofl

**Table 8:** Distribution of antibiotic resistance genes in strains of *E. coli* isolated from chicken.

Antimicrobial Agent	Phenotypic Resistance	Resistance Gene	No. of Positive Isolates (%)
Tetracycline	230	*tetA*	126 (54.8)
		*tetB*	119 (51.7)
Sulfadiazine	230	*sul1*	115 (50.0)
Streptomycin	216	*aadA1*	63 (29.2)
Ciprofloxacin	39	*qnrA*	0

## Data Availability

All the data presented in the study are included in the article; further inquiries can be directed to the corresponding authors.
